# Cobalt Oxide-Decorated Silicon Carbide Nano-Tree Array Electrode for Micro-Supercapacitor Application

**DOI:** 10.3390/ma14164514

**Published:** 2021-08-11

**Authors:** Chuan-Pei Lee, Bayu-Tri Murti, Po-Kang Yang, Francesca Rossi, Carlo Carraro, Roya Maboudian

**Affiliations:** 1 Department of Applied Physics and Chemistry, University of Taipei, Taipei 10048, Taiwan; d96524014@ntu.edu.tw; 2 Berkeley Sensor & Actuator Center, Department of Chemical and Biomolecular Engineering, University of California, Berkeley, CA 94720, USA; carraro@berkeley.edu; 3 Graduate Institute of Biomedical Materials and Tissue Engineering, College of Biomedical Engineering, Taipei Medical University, Taipei 11031, Taiwan; 4 Department of Biomedical Sciences and Engineering, National Central University, Chung-li 32001, Taiwan; pkyang@ncu.edu.tw; 5 IMEM-CNR Institute, Parco Area delle Scienze 37/A, 43124 Parma, Italy; frossi@imem.cnr.it

**Keywords:** chemical vapor deposition, cobalt oxide, micro-supercapacitor, nanowire, silicon carbide

## Abstract

A cobalt oxide (Co_3_O_4_)-decorated silicon carbide (SiC) nano-tree array (denoted as Co_3_O_4_/SiC NTA) electrode is synthesized, and it is investigated for use in micro-supercapacitor applications. Firstly, the well-standing SiC nanowires (NWs) are prepared by nickel (Ni)-catalyzed chemical vapor deposition (CVD) method, and then the thin layer of Co_3_O_4_ and the hierarchical Co_3_O_4_ nano-flower-clusters are, respectively, fabricated on the side-walls and the top side of the SiC NWs via electrodeposition. The deposition of Co_3_O_4_ on the SiC NWs benefits the charge transfer at the electrode/aqueous electrolyte interface due to its extremely hydrophilic surface characteristic after Co_3_O_4_ decoration. Furthermore, the Co_3_O_4_/SiC NTA electrode provides a directional charge transport route along the length of SiC nanowires owing to their well-standing architecture. By using the Co_3_O_4_/SiC NTA electrode for micro-supercapacitor application, the areal capacitance obtained from cyclic voltammetry measurement reaches 845 mF cm^−2^ at a 10 mV s^−1^ scan rate. Finally, the capacitance durability is also evaluated by the cycling test of cyclic voltammetry at a high scan rate of 150 mV s^−1^ for 2000 cycles, exhibiting excellent stability.

## 1. Introduction

Planar on-chip micro-supercapacitors are used as compact microscale, integrated, and reliable power sources for advanced microelectronics; meanwhile, they could also be integrated with microelectromechanical devices [1,2]. To date, various planar micro-supercapacitors have been studied in several papers [1,2,3,4,5,6,7,8,9]; the device-level integration of the electrode material is the major challenge of such devices. Among the electrode materials, carbonaceous materials are most commonly used for micro-supercapacitors, such as activated carbon [8,10], carbon nanotubes [3] and graphene [11], etc. However, these carbon-based materials are usually hydrophobic and result in poor surface wetting toward aqueous electrolytes; however, aqueous electrolytes are more environmentally friendly, stable, low-cost, and biocompatible than organic-based electrolytes. Accordingly, a promising electrode material, namely silicon carbide (SiC), has recently attracted considerable global interest in the field of aqueous electrolyte-based micro-supercapacitors, since it consists of abundant elements of earth and it has several excellent natures, such as superior durability under high current density and in various harsh conditions, high chemical stability and high mechanical strength [12,13]. Most importantly, it is more hydrophilic than the pristine carbonaceous materials mentioned above [14,15,16]. On the other hand, it is well known that electrode materials having one-dimensional (1-D) nanostructure could provide extremely high specific surface areas, 1-D directional charge transport route, sufficient space for ion transfer, and structural stability during the charge/discharge processes [12,17,18,19,20]; those properties are desirable in capacitor applications. Accordingly, Chen et al. utilized a carbothermal reduction method to prepare SiC nanowire (NW) film on graphite paper to test its capacitive behavior [21]. The electrode with SiC nanowire film on graphite paper exhibits a specific capacitance of 37 mF cm^−2^ at 0.3 A cm^−2^, and it also shows excellent capacity retention (i.e., 100%) after 2000 cycles. In our group, Alper et al. synthesized well-standing SiC NWs directly on a SiC/SiO_2_/Si(100) substrate as a highly robust electrode for micro-supercapacitor application [22]. Although the SiC NW electrode shows exceptional durability, i.e., 95% capacitance retention after 2 × 10^5^ charge/discharge cycles at 5 V s^−1^ scan rate, its low areal capacitance of ~240 µF cm^−2^ measured at 100 mV s^−1^ still requires further improvement.

In this work, Co_3_O_4_ thin layer and hierarchical Co_3_O_4_ nano-flower-clusters are, respectively, fabricated on the side walls and the top side of the SiC NWs via the electrodeposition technique. The areal capacitance of the as-synthesized Co_3_O_4_/SiC nano-tree array (denoted as Co_3_O_4_/SiC NTA) electrode can reach 845 mF cm^−2^ at 10 mV s^−1^ scan rate; its durability is also evaluated by cyclic voltammetry for 2000 cycles at a high scan rate of 150 mV s^−1^. Furthermore, the decoration of Co_3_O_4_ on SiC NWs not only provides high electrochemical activity, but also a low water contact angle that could remarkably improve the interface contact between electrode and aqueous electrolytes.

## 2. Materials and Methods

### 2.1. Synthesis of SiC NWs

The n-type 4H-SiC(0001) (Cree Research, Silicon Drive, NC, USA) wafer was used as the substrate for growing the SiC NWs on it. The 4H-SiC(0001) wafer were carefully cleaned before use, and the SiC NW arrays were prepared on the abovementioned substrate according to our previous reports, i.e., the Ni-catalyzed CVD method [12,22,23]. In brief, a ~2.5 nm Ni film was fabricated onto a cleaned 4H-SiC(0001) wafer substrate by using the electron beam evaporation; the as-prepared substrate was then transported into a hot-wall chemical vapor deposition (CVD) tube furnace to a base pressure of 30 × 10^−3^ Torr, wherein it was further heated to 950 °C at a rate of 50 °C min^−1^ at ~5 Torr under 10 sccm flow of H_2_ (Praxair, 99.99%, Danbury, CT, USA). Once the furnace reached 950 °C, methyltrichlorosilane (MTS, Sigma-Aldrich, Saint Louis, MO, USA) was introduced into the reactor (0.5 sccm, 1 h); after that, the MTS source was turned off and the tube furnace was cooled down to ambient temperature under 10 sccm H_2_ flow. The 4H-SiC(0001) wafer/SiC NWs electrode, denoted as the SiC NW array electrode hereafter, was thus prepared for this study.

### 2.2. Co_3_O_4_ Deposition

The Co_3_O_4_ deposition processes were carried out according to the previous report [12]. The Co_3_O_4_ was electro-deposited on the SiC NWs by using an electrochemical workstation (CH Instruments Inc., 660D, Bee Cave, TX, USA) at −0.9 V for 10 min in a three-electrode system. Before electro-deposition, the SiC NW array electrode was immersed in an isopropanol solution containing 1 M Cobalt(II) sulfate hydrate (CoSO_4_·xH_2_O; F.W. 155, Aldrich, USA) overnight and then rinsed with deionized water (DI-Water) and isopropanol (IPA, Sigma-Aldrich, USA) sequentially, and then dried by using N_2_ gas. The as-pretreated SiC NW array electrode, a Ag/AgCl/saturated KCl(aq) and a Pt foil were used as the working, reference, and counter electrodes, respectively. The electro-deposition of Co_3_O_4_ was performed in 1 M CoSO_4_·xH_2_O aqueous electrolyte. Finally, the Co_3_O_4_-deposited electrode was sintered under 350 °C for 1 h in air atmosphere. The Co_3_O_4_/SiC NTA electrode was thus prepared for this study.

### 2.3. Materials Characterization

The field emission scanning electron microscopy (FE-SEM, Nova Nano-SEM 230, FEI, Hillsboro, OR, USA) was utilized to observe the morphologies of the as-synthesized electrodes. A Horiba Jobin Yvon LabRam confocal Raman system (Horiba Jobin Yvon LabRam, excitation line provided by a HeNe laser at 632.8 nm, through a 100× objective with 0.8 numerical aperture) was used for Raman measurement. A JEOL-2100 transmission electron microscope (JEOL Ltd., Tokyo, Japan) was utilized to obtain the high-resolution transmission electron microscopy (HRTEM) images and the selected area electron diffraction (SAED) spectra under an acceleration voltage of 200 kV. The elemental distribution of Si, Co, and O in the Co_3_O_4_/SiC NTA was analyzed by energy dispersive X-ray spectroscopy (EDS) equipped on the TEM.

### 2.4. Electrochemical Characterization

Electrochemical measurements were carried out by using a potentiostat/galvanostat (PGSTAT 30, Autolab, Eco-Chemie, Utrecht, The Netherlands) with a three–electrode electrochemical cell containing an aqueous electrolyte of 5 M KOH. The as-pretreated Co_3_O_4_/SiC NTA electrode, a platinum sheet, and a saturated calomel electrode (SCE) reference electrode were used as the working electrode, counter electrode, and reference electrode, respectively. The areal capacitance was investigated by using cyclic voltammetry measurement at various scan rates [24].

## 3. Results and Discussion

**Figure 1:**
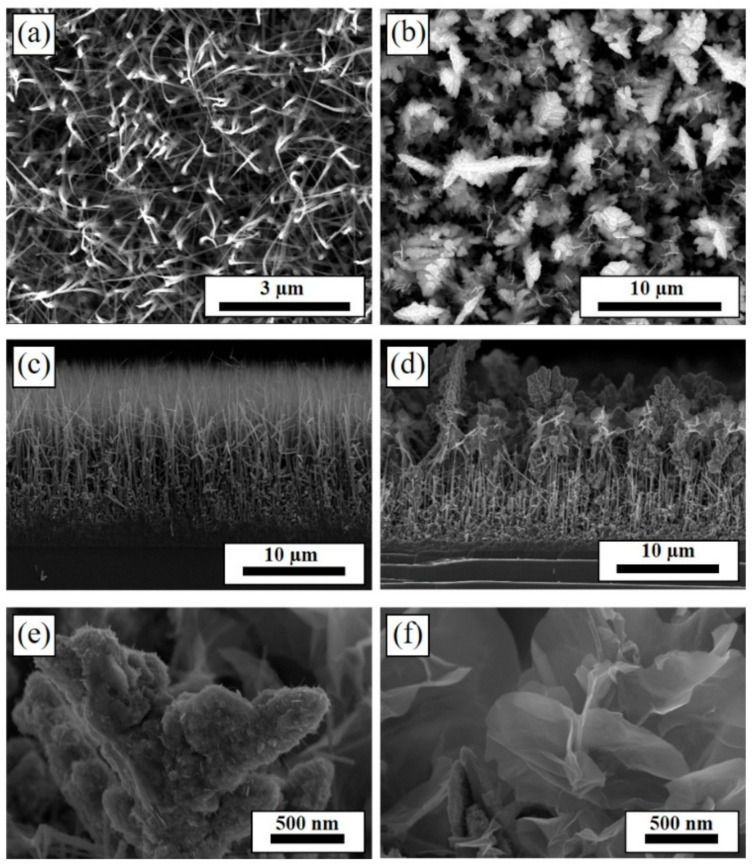
The top-view SEM images of the SiC NW film (**a**) before and (**b**) after Co_3_O_4_ decoration; their corresponding cross-section SEM images are shown in (**c**,**d**), respectively. (**e**,**f**) Are the high-magnification cross-section SEM images of the top side of (**d**).

**Figure 2:**
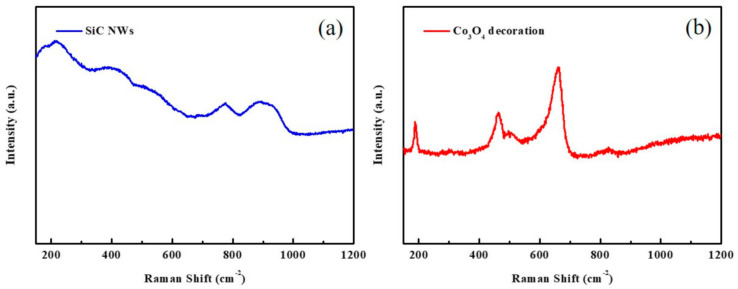
Raman spectra of (**a**) SiC NW film, and (**b**) Co_3_O_4_/SiC NTA film.

The well-standing silicon carbide nanowires (designated as SiC NWs) were synthesized on SiC wafer substrates by a Ni-catalyzed CVD method, and then the Co_3_O_4_ was decorated on the SiC NWs to form Co_3_O_4_/SiC nano-tree arrays (denoted as Co_3_O_4_/SiC NTAs) via the electrodeposition technique. The as-prepared SiC NW film and Co_3_O_4_/SiC NTA film are first observed by field emission scanning electron microscopy (FE-SEM), as shown in Figure 1. Figure 1a,b show the top view SEM images of the SiC NW film and Co_3_O_4_/SiC NTA film, respectively. It can be seen that the top side of SiC NWs looks like a needle tip structure; after the electrodeposition of Co_3_O_4_, the hierarchical Co_3_O_4_ nano-flower-clusters appear on the top side of SiC NWs. The cross-section SEM images of above two samples are shown in Figure 1c,d. As shown in Figure 1c, the SiC NWs have directly grown on the substrate, and the thickness of the SiC NW film was around 15~17 μm. Furthermore, the SiC NW film is consisted of needle-like nanostructure with root diameter of ~230 nm at the bottom-side and ~10 nm at the top-side. In Figure 1d, we can obviously find that the top side of the SiC NWs was deposited with hierarchical Co_3_O_4_ nano-flower-clusters with irregular sizes and shapes. The high-magnification SEM images focusing on the partial region of Co_3_O_4_ nano-flower clusters from a cross-section view are shown in Figure 1e,f. In Figure 1e, the morphology shows a hierarchical structure with rough surface. In Figure 1f, a flower-like morphology is shown consisting of sheet structures. The as-prepared SiC NW film and Co_3_O_4_/SiC NTA film were further characterized by Raman spectroscopy (Figure 2). As shown in Figure 2a, the SiC NW film shows a hexagonal crystal structure composed of polytypes of SiC, and is predominantly 4H-like [23]. In Figure 2b, three sharp peaks at 658, 460, and 185 cm^−1^ were detected in the Raman spectrum of Co_3_O_4_/SiC NTA film; those peaks correspond to 1 A_1g_, 1 E_g_ and 3 F_2g_ Raman active modes of Co_3_O_4_ [12,25,26]. As indicated in the previous report published by G. Varga et al. [27], both the Raman (Figure 2b) and X-ray diffraction (XRD, Figure S1 in the Supporting Information) measurements imply that a mixed spinel oxide structure could be produced via our synthesis route.

**Figure 3:**
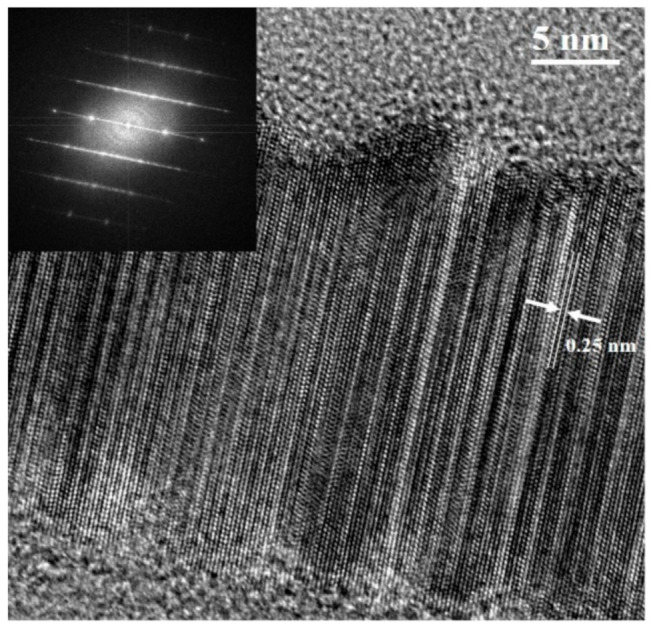
HRTEM image of a SiC NW with its corresponding selected area electron diffraction pattern (inset).

**Figure 4:**
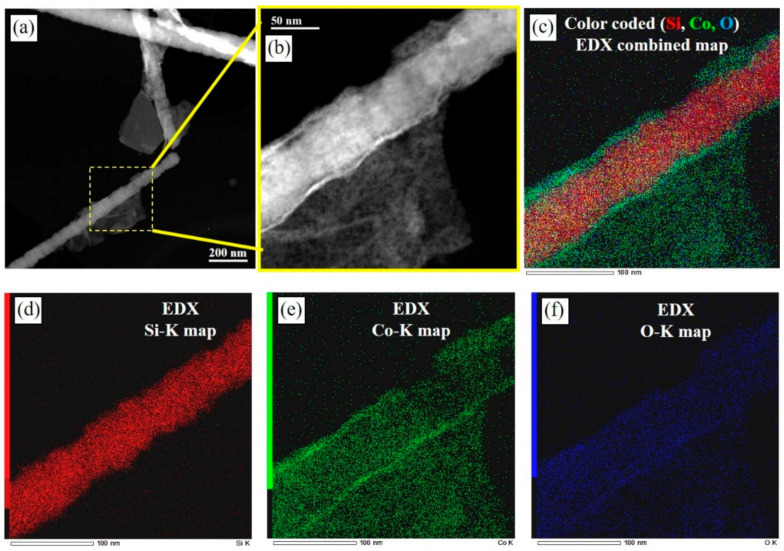
Selected TEM image of (**a**) the Co_3_O_4_-decorated SiC NWs with (**b**) Co_3_O_4_ nano-flower-clusters; the corresponding mapping image of (**b**) is shown in (**c**), and the individual elemental Si (red), Co (green), and O (blue) mapping images are shown in (**d**–**f**), respectively.

The HRTEM and EDS are further utilized to characterize the samples, as shown in Figure 3 and Figure 4. Figure 3 shows the lattice fringe of a SiC NW, where a lattice spacing of about 0.25 nm can be assigned to the <111> lattice plane of SiC (i.e., along the c-axis of hexagonal polytypes or the <111> direction of the cubic polytype) [22,28]. As shown in the inset of Figure 3a, the typical streaking of reflections in this Fourier transform image confirm the presence of stacking faults along the NW axis; a high density of stacking faults associated with the presence of poly-types of SiC (i.e., mainly 3C-, 4H- and 6H-SiC). Figure 4a shows the TEM image of the Co_3_O_4_-decorated SiC NWs with Co_3_O_4_ sheets, and the enlarged image of the region in yellow dash frame is shown in Figure 4b. As shown in Figure 4b, the Co_3_O_4_ coating on the side-wall of SiC NW is conformal and continuous, and the thickness of the Co_3_O_4_ coating layer is around 7~18 nm. Moreover, the conformal and continuous characters of the Co_3_O_4_ coating is further verified by the elemental distribution maps (Figure 4c–f) for a representation of the image shown in Figure 4b. Both Co and O characteristics of the Co_3_O_4_ are well distributed over the surface of SiC NW, revealing that the electrodeposition is effective for material loading onto high aspect-ratio nanostructures. We expect that the coating of Co_3_O_4_ layer on the surface of SiC NWs could distribute the charge flux evenly along the one-dimensional nanostructure of the SiC NWs, and the effective utilization of the high surface area of the SiC NWs would lead to less charge accumulation; both would facilitate the charge transfer kinetics in the electrochemical reaction.

**Figure 5:**
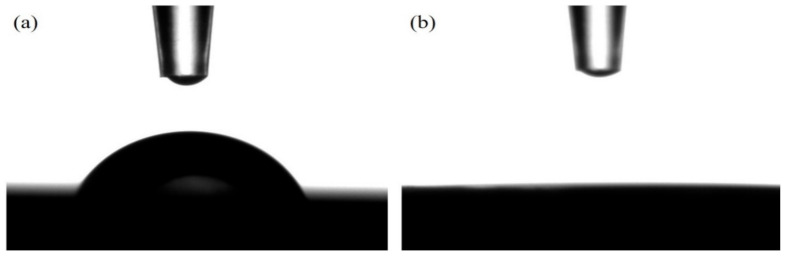
Surface contact angles of water droplets on the SiC NW film (**a**) before and (**b**) after Co_3_O_4_ decoration.

On the other hand, a zero-contact angle toward water droplets was observed on the SiC NW film was Co_3_O_4_ decoration, whereas the pristine SiC NW film shows a contact angle of ~60° (see Figure 5). The above results reveal that the water droplet can completely wet the surface of Co_3_O_4_/SiC NTA electrode and the surface is extremely hydrophilic, which would benefit the charge transfer at the interface of the aqueous electrolyte and the Co_3_O_4_/SiC NTA electrode. This advantage can be supported by the report [17], which demonstrated that the electrode exhibited lower contact angle toward water droplet usually possesses lower charge transfer resistance at the interface between the electrode and the aqueous electrolyte.

**Figure 6:**
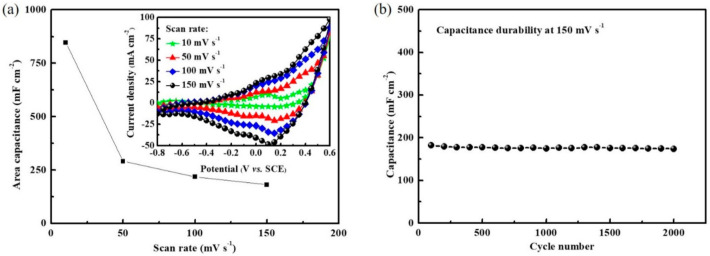
(**a**) Variation of areal capacitance for scan rate by using the Co_3_O_4_/SiC NTA electrode, which was obtained from the cyclic voltammetry curves, as shown in the inset. (**b**) The capacitance durability of Co_3_O_4_/SiC NTA electrode measured at 150 mV s^−1^.

Figure 6a shows the variation of areal capacitance for different scan rates by using a Co_3_O_4_/SiC NTA electrode. All the areal capacitance values are extracted from the cyclic voltammetry curves, as shown in the inset of Figure 6a. As the scan rate increased, the current density increased; all cyclic voltammetry curves remain of similar shape even at high scan rates, suggesting that the inner area underneath the Co_3_O_4_/SiC NTA was able to contribute to the capacitance because of the hierarchical structure (i.e., the Co_3_O_4_ nano-flower-clusters and continuous/conformal Co_3_O_4_ coating on SiC NW arrays) developed inside the Co_3_O_4_/SiC NTA electrode. The areal capacitance values were calculated to be 845, 291, 218, and 182 mF cm^−2^ at scan rates of 10, 50, 100, and 150 mV s^−1^, respectively. To evaluate the durability of the Co_3_O_4_/SiC NTA electrode, the cycling performance was tested under a high scan rate of 150 mV s^−1^, and the obtained capacitance values in each cycle were plotted as a function of the cycle number (Figure 6b). Herein, we observed a negligible change on the capacitance of the Co_3_O_4_/SiC NTA electrode after 2000 cycles.

## 4. Conclusions

In summary, we synthesize an efficient Co_3_O_4_/SiC NTA electrode for application in micro-supercapacitors. By using the electrodeposition technique, a Co_3_O_4_ thin layer and hierarchical Co_3_O_4_ nano-flower clusters can be successfully decorated on the side walls and the top side of the SiC NWs, respectively. The areal capacitance of the as-prepared Co_3_O_4_/SiC NTA electrode can reach 845 mF cm^−2^ at 10 mV s^−1^; moreover, its durability is also evaluated by the cycling test of cyclic voltammetry for 2000 cycles at a high scan rate of 150 mV s^−1^. As compared to the previous report using the SiC NW-based electrode [21], the good performance of the Co_3_O_4_/SiC NTA electrode arises from the synergetic effects between Co_3_O_4_ and SiC NW arrays. The SiC NW arrays provide a directional charge transport route along the nanowire length in the Co_3_O_4_/SiC NTA electrode, as well as high surface area for Co_3_O_4_ catalyst loading. Furthermore, the electro-deposited Co_3_O_4_ provides high electrochemical activity, and exhibits zero contact angle toward aqueous solutions that facilitates the interfacial contact between electrode and aqueous electrolyte. The concurrent advantage of earth-abundant constituent materials, good performance, and durability could make the Co_3_O_4_/SiC NTA electrode a viable candidate for micro-supercapacitor mass production.

## Data Availability

Data is available within the article.

## Supplementary Materials

The following are available online at https://www.mdpi.com/article/10.3390/ma14164514/s1, Figure S1: The XRD (X-ray diffraction) patterns of the Co_3_O_4_ powder collected form the Co_3_O_4_/SiC NTA electrodes via ultrasonic. (a) Before and (b) after the cycling test of cyclic voltammetry.
