# Supplementary material for: Cobalt Oxide-Decorated Silicon Carbide Nano-Tree Array Electrode for Micro-Supercapacitor Application
Source: Materials (Basel). 2021 Aug 11;14(16):4514. doi: 10.3390/ma14164514 (PMC8400218; doi:10.3390/ma14164514)
Supplement: Supplementary file 1 [file materials-14-04514-s001.zip › materials-1296470 -supplementary.pdf]

materials

Article

Cobalt Oxide-Decorated Silicon Carbide Nano-Tree Array Electrode for Micro-Supercapacitor Application

Chuan-Pei Lee [1,2,*], Bayu-Tri Murti [1,3,4], Po-Kang Yang [4], Francesca Rossi [5], Carlo Carraro [2] and Roya Maboudian [2,*]

1 Department of Applied Physics and Chemistry, University of Taipei, Taipei 10048, Taiwan; CPLee@utaipei.edu.tw
2 Department of Chemical and Biomolecular Engineering, Berkeley Sensor &Actuator Center, University of California, Berkeley, CA 94720, USA; carraro@yahoo.com
3 Graduate Institute of Biomedical Materials and Tissue Engineering, College of Biomedical Engineering, Taipei Medical University, Taipei 11031, Taiwan
4 Department of Biomedical Sciences and Engineering, National Central University, Chung-li 32001, Taiwan; pkyang@ncu.edu.tw
5 IMEM-CNR Institute, Parco Area delle Scienze 37/A, 43124 Parma, Italy; frossi@imem.cnr.it
* Correspondence: CPLee@utaipei.edu.tw (C.-P.L.); maboudia@berkeley.edu (R.M.)

Abstract: A cobalt oxide (Co_3O_4)-decorated silicon carbide (SiC) nano-tree array (denoted as Co_3O_4/SiC NTA) electrode is synthesized, and it is investigated for the use in the micro-supercapacitor. Firstly, the well-standing SiC nanowires (NWs) are prepared by nickel (Ni)-catalyzed chemical vapor deposition (CVD) method, and then the thin layer of Co_3O_4 and the hierarchical Co_3O_4 nanoflower-clusters are, respectively fabricated on the side-walls and the top side of the SiC NWs via electrodeposition. The deposition of Co_3O_4 on the SiC NWs benefits for the charge transfer at the electrode/aqueous electrolyte interface due to its extremely hydrophilic surface characteristic after Co_3O_4 decoration. Furthermore, the Co_3O_4/SiC NTA electrode would possess a directional charge transport route along the nanowire length of SiC NWs owing to their well-standing architecture. By using the Co_3O_4/SiC NTA electrode for micro-supercapacitor, the areal capacitance obtained from cyclic voltammetry measurement reaches 845 mF cm^{-2} at a 10 mV s^{-1} scan rate. Finally, the capacitance durability is also evaluated by the cycling test of cyclic voltammetry at a high scan rate of 150 mV s^{-1} for 2000 cycles.

Keywords: chemical vapor deposition; cobalt oxide; micro-supercapacitor; nanowire; silicon carbide

Citation: Lee, C.-P.; Murti, B.-T.; Yang, P.-K.; Rossi, F.; Carraro, C.; Maboudian, R. Cobalt Oxide-Decorated Silicon Carbide Nano-Tree Array Electrode for Micro-Supercapacitor Application. *Materials* **2021**, *14*, 4514. https://doi.org/10.3390/ma14164514

Academic Editor: Christian M. Julien

Received: 28 June 2021
Accepted: 02 August 2021
Published: 11 August 2021

Publisher's Note: MDPI stays neutral with regard to jurisdictional claims in published maps and institutional affiliations.

Copyright: © 2021 by the authors. Submitted for possible open access publication under the terms and conditions of the Creative Commons Attribution (CC BY) license (http://creativecommons.org/licenses/by/4.0/).

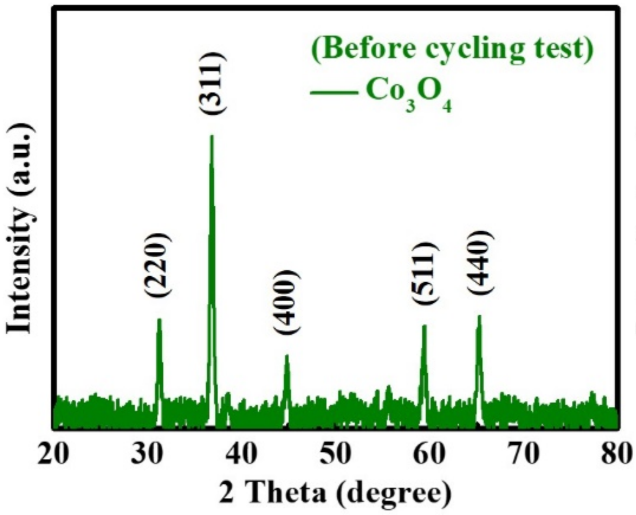

(**a**)

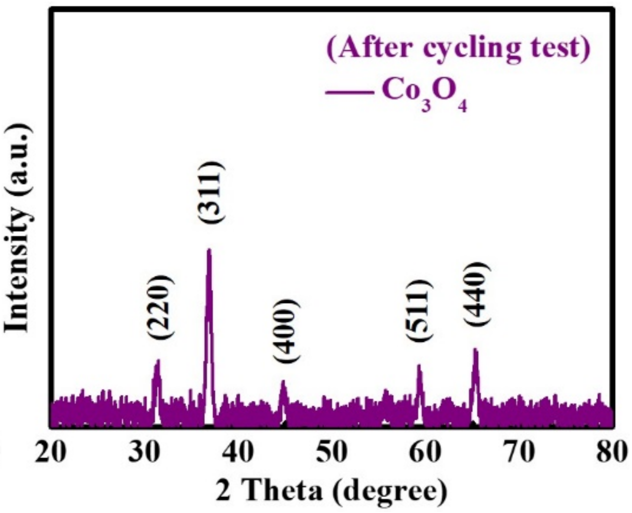

(**b**)

Figure S1. The XRD (X-ray diffraction) patterns of the Co_3O_4 powder collected form the Co_3O_4/SiC NTA electrodes via ultrasonic. (**a**) Before and (**b**) after the cycling test of cyclic voltammetry.

Figure S1 shows the XRD (X-ray diffraction) patterns of the Co_3O_4 powder collected form the Co_3O_4/SiC NTA electrodes before and after the cycling test of cyclic voltammetry. As revealed in Figure S1, all the diffraction peaks are attributed to spinel Co_3O_4 phase (JCPDS 42-1467), indicating that the crystalline Co_3O_4 has been formed as the Co_3O_4-deposited electrode was sintered under 350 °C for 1 h in air atmosphere; moreover, the crystalline structure of Co_3O_4 is still remaining after the cycling test of cyclic voltammetry.
